# Treatment of Non-Anastomotic Biliary Strictures after Liver Transplantation: How Effective Is Our Current Treatment Strategy?

**DOI:** 10.3390/jcm12103491

**Published:** 2023-05-16

**Authors:** Florian A. Michael, Mireen Friedrich-Rust, Hans-Peter Erasmus, Christiana Graf, Olivier Ballo, Mate Knabe, Dirk Walter, Christoph D. Steup, Marcus M. Mücke, Victoria T. Mücke, Kai H. Peiffer, Esra Görgülü, Antonia Mondorf, Wolf O. Bechstein, Natalie Filmann, Stefan Zeuzem, Jörg Bojunga, Fabian Finkelmeier

**Affiliations:** 1 Department of Internal Medicine 1, University Hospital Frankfurt, Goethe University, Theodor-Stern-Kai 7, 60590 Frankfurt, Germany; 2 Department of General and Visceral Surgery, University Hospital Frankfurt, Goethe University, Theodor-Stern-Kai 7, 60590 Frankfurt, Germany; 3 Institute of Biostatistics and Mathematical Modeling, Goethe University, Theodor-Stern-Kai 7, 60590 Frankfurt, Germany

**Keywords:** ischemic-type biliary lesion, endoscopy, stenting, balloon dilatation, endoscopic retrograde cholangiopancreatography

## Abstract

Background: Non-anastomotic biliary strictures (NAS) are a common cause of morbidity and mortality after liver transplantation. Methods: All patients with NAS from 2008 to 2016 were retrospectively analyzed. The success rate and overall mortality of an ERCP-based stent program (EBSP) were the primary outcomes. Results: A total of 40 (13.9%) patients with NAS were identified, of which 35 patients were further treated in an EBSP. Furthermore, 16 (46%) patients terminated EBSP successfully, and nine (26%) patients died during the program. All deaths were caused by cholangitis. Of those, one (11%) patient had an extrahepatic stricture, while the other eight patients had either intrahepatic (3, 33%) or combined extra- and intrahepatic strictures (5, 56%). Risk factors of overall mortality were age (*p* = 0.03), bilirubin (*p* < 0.0001), alanine transaminase (*p* = 0.006), and aspartate transaminase (*p* = 0.0003). The median duration of the stent program was 34 months (ITBL: 36 months; IBL: 10 months), and procedural complications were rare. Conclusions: EBSP is safe, but lengthy and successful in only about half the patients. Intrahepatic strictures were associated with an increased risk of cholangitis.

## 1. Introduction

Biliary complications are a common cause of morbidity and mortality following liver transplant. Biliary strictures can be distinguished into anastomotic and non-anastomotic biliary strictures (NAS) [1,2].

Anastomotic strictures usually occur limited to the biliary anastomosis between the donor and the recipient bile duct. They are caused by fibrotic healing of a short segment, narrowing the anastomosis [3]. The frequency of anastomotic strictures was about 13% in a meta-analysis, with a higher risk in living donor organ transplants (19%) compared to deceased donor organ transplants (12%). In 88% of patients, duct-to-duct anastomosis was performed with regular anatomy of the gastrointestinal tract [4]. Therefore, repeat stenting via endoscopic retrograde cholangiopancreatography (ERCP) is the first-line approach with a described success rate of 57–100% [3,4,5,6,7]. Alternative options are percutaneous transhepatic biliary drainage (PTBD), especially in patients with a bilioenteric anastomosis with a Roux-en-Y loop. If interventional methods fail, surgical reconstruction of the bile duct by bilioenteric anastomosis is the next step. As ultima ratio, a second liver transplantation can be performed in cases of liver failure or recurrent cholangitis [2].

The characterization of NAS is less defined in the literature. NAS can be described as one or more strictures and/or dilatations of the bile ducts proximal to a biliary anastomosis. Typically, the strictures are longer, at multiple sites, and they occur 3–6 months after liver transplantation, which is earlier than with anastomotic strictures [2].

There are two different subtypes of NAS: ischemic biliary lesions (IBLs) caused by a thrombosis in the hepatic artery, and ischemic-type biliary lesions (ITBLs) [8]. The etiology of ITBLs is multifactorial and includes ischemia/reperfusion injury, immunologically mediated injury, and bile salt-mediated injury of the biliary epithelium and duct walls [1]. NAS is classified into three types: extrahepatic lesions (type I), intrahepatic lesions (type II), and combined intra- and extrahepatic lesions (type III) [9].

In comparison to anastomotic strictures, endoscopic approaches to treat NAS have only been examined by few retrospective trials [8,9,10,11,12]. The aim of the present study was to evaluate the efficacy and safety of endoscopic treatment strategies in NAS at a German tertiary care hospital and liver transplant center.

## 2. Materials and Methods

The present study was designed as a retrospective single-center study conducted at the Department of Internal Medicine 1 at the University Hospital in Frankfurt, Germany. The study was approved by the Institutional Review Board of Frankfurt Goethe University (365/17). All research was conducted in accordance with both the Declarations of Helsinki and Istanbul.

- Definitions

NAS was defined as one or multiple strictures in the biliary tract system not related to the anastomotic region following liver transplantation [13]. NAS was divided into ITBL and IBL. IBL was defined as one or more strictures occurring after an occlusion of the hepatic artery following transplantation [13]. ITBL was defined as one or more strictures not related to a previous occlusion of the hepatic artery [14].

After liver transplantation, all patients underwent a follow-up focusing on clinical conditions, changes in laboratory values, and sonographic findings. As indicated, a computed tomography scan or a magnetic resonance imaging with contrast agent during the arterial phase was performed to confirm occlusion of the hepatic artery, thereby confirming IBL with consecutive surgery to regain arterial perfusion.

All NAS cases were classified by the ERCP reports into extrahepatic (type I), intrahepatic (type II), or combined extra- and intrahepatic strictures (type III) [15]. Progressive strictures were reclassified during the ERCP-based stent program if appropriate. Currently, the severity of ITBL is not defined uniformly in the literature. Therefore, the severity of strictures was assessed by the endoscopists performing the procedure into either persisting or resolved biliary strictures. If ERCP showed normalized drainage, the endoscopic stent program was terminated. These patients underwent further clinical and sonographic follow-up every 3–6 months.

In this study, the phrase “ERCP-based stent program” is defined as all endoscopic stenting treatments performed in each patient with NAS after liver transplantation until successful resolution of NAS with removal of all stents, switch to a surgical approach, death, or end of follow-up (31 May 2020). In cases of NAS being diagnosed in patients after liver transplant receiving an ERCP due to other reasons, previous ERCPs since transplant were retrospectively analyzed for unrecognized signs of NAS. The duration of ERCP-based stent programs was measured from the first ERCP with signs of NAS to program termination due to any of the abovementioned reasons. The total number of ERCP procedures and the number of ERCP procedures since diagnosis of NAS were counted. The biliary stents placed were plastic stents (Biliary endoprosthesis, type double pigtail, size 7Fr to 10Fr, Optimed, Ettlingen, Germany) or metal stents (Nitri-S^TM^ Stent, TaeWoong Medical, Gimpo-si, Republic of Korea).

The occurrence of cholangitis and pancreatitis was monitored, among other complications. Cholangitis was defined according to the Tokyo guidelines as a combination of systematic inflammation, cholestasis, and appropriate imaging [16]. Pancreatitis was defined according to the revised Atlanta classification. Two or more of the following criteria were required to be met for the diagnosis of acute pancreatitis: (a) abdominal pain suggestive of pancreatitis, (b) serum amylase or lipase level greater than three times the upper normal value, or (c) characteristic imaging findings [2]. Severity was classified as mild, moderate, or severe depending on local complications and organ failure [17].

- Inclusion and exclusion criteria for the analysis of the ERCP-based stent program

A systematic search of available electronic patient files using the terms “ischemic-type biliary lesion”, “ischemic biliary lesion”, “ITBL”, “IBL”, “non-anastomotic biliary strictures”, and “NAS” was performed to identify patients eligible for inclusion. Patients were screened from 2008 to 2016.

Included were all patients who (I) underwent liver transplantation and (II) developed NAS (either IBL or ITBL) with (III) subsequent ERCP-based stent program at our hospital.

Exclusion criteria were (I) patients not fulfilling all inclusion criteria or not undergoing the entire stent program at our hospital, (II) patients with strictures other than NAS, (III) patients with isolated anastomotic stricture without simultaneous NAS, (IV) patients who underwent surgical management of NAS either before or instead of ERCP, except for prior surgical revascularization of the hepatic artery.

- Endpoints

First, all procedures and outcomes were collected from selected patients diagnosed with NAS after liver transplantation who met the study criteria.

Then, the procedural outcomes and success rates and peri-interventional data such as duration and number of performed ERCPs, change in treatment approach, technical parameters, and complication rates of patients who underwent an ERCP-based stent program as the first-line approach were analyzed. All patients were further subdivided into those with ITBL vs. IBL and further by location of strictures (type I to III).

Lastly, regression analysis of baseline variables and laboratory values was performed, assessing successful termination of the ERCP-based stent program and overall mortality.

- Statistical methods

Statistical analysis was performed using IBM SPSS Statistics version 21 (IBM Corp. Somers, New York, NY, USA) and R (version 4.0.4, R Core Team (2021), Vienna, Austria).

Descriptive statistics were computed to provide frequencies for categorical variables and median with 25th and 75th percentiles for continuous values.

Laboratory values were included as time-dependent variables, and death was defined as competing risk using the Aalen–Johansen estimator.

Univariate Cox regression and univariate competing risk regression were used to find predictive factors for overall mortality and successful termination of the ERCP-based stent program. The Cox proportional regression model assumes a linear relationship between the endpoint and quantitative predictor variables. Predicator variables that have a highly skewed distribution, as expected by the laboratory values, may require logarithmic transformation to reduce the effect of extreme values. A survival analysis was performed using Kaplan–Meier survival analysis and Cox regression analysis.

Results were expressed as hazard ratios (HRs) with 95% confidence intervals (CIs). The test for the influence of laboratory values was one-sided, while all other tests were two-sided; *p*-values ≤ 0.05 were considered statistically significant.

## 3. Results

A total of 287 patients underwent liver transplantation between 2008 to 2016. Of these patients, 40 patients (13.9%) were diagnosed with NAS (ITBL: 32, 11.1%; IBL: 8; 2.2%). Follow-up was ended in May 2020.

Median time to diagnosis of NAS, ITBL, and IBL was 5, 6, and 3 months after liver transplantation, respectively.

- Descriptive analysis of all patients with NAS

One patient underwent a single ERCP with stent exchange because of cholangitis but was lost to follow-up afterward. A total of 35 patients (88%) were treated in an ERCP-based stent program, and four (10%) were treated primarily with a bilioenteric anastomosis. Two of the four surgically treated patients underwent an additional PTBD program. Three of the four patients needed re-transplantation, of which two survived. One patient was successfully treated by a combination of bilioenteric anastomosis and PTBD program.

Of the 35 patients primarily treated in the ERCP-based stent program, 16 patients (41%) successfully finished endoscopic stenting without needing a different treatment approach, five patients (13%) were still undergoing endoscopic stenting at the end of follow-up, five patients (13%) switched to a surgical approach, and three of them (9%) switched to PTBD treatment. Supplementary Figure S1 renders an overview of the procedural paths of all patients included in the present trial.

Cholangitis was the cause of death in all nine patients (23%) who died during the ERCP-based stent program. Organ transplantation was indicated in all these patients. However, one patient refused further treatment; one patient was not listed again for liver transplantation because of their expressed wish against transfusion of blood products; four patients had contraindications to transplantation (three with septic shock and one with a severe critical illness myopathy); lastly, three patients did not receive an organ in time. Supplementary Table S1 displays the specific data of the nine deceased patients.

- Analysis of the ERCP-based stent program

**Figure 1:**
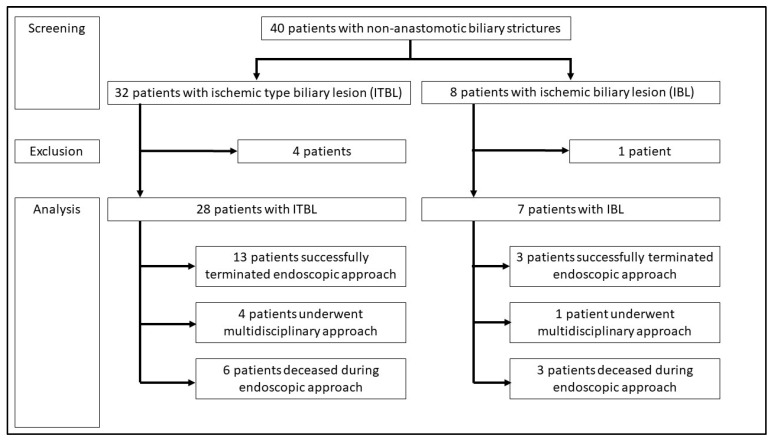
Flowchart of patient analysis undergoing ERCP-based stent program in the present trial.

In one subgroup, only patients in the ERCP-based stent program were analyzed, including 35 of 40 (88%) patients (ITBL: 28 patients, 70%; IBL: seven patients, 18%). One patient who only received a single stent replacement and four patients who had primary surgical treatment were excluded (ITBL: 4, IBL: 1). Figure 1 displays a flowchart of screening, exclusion, and analysis of the patients undergoing ERCP-based stent program. Table 1 presents the baseline and surgical characteristics of all included patients.

All 35 patients who were in the ERCP-based stent program to treat NAS received a first ERCP including endoscopic papillotomy and cholangiography to visualize the biliary system and classify NAS into type I to III. In all patients, a stent placement was performed after lavage of the biliary system: 28 (80%) received single stent placement, whereas two stents were placed (usually one into each right and left bile duct) in seven patients (20%).

According to the medical report, the major stricture was successfully stented in 30 patients (85%) after the first ERCP.

In 15 (43%) patients, additional balloon dilatation was performed at least once during the ERCP-based stent program. Casts were extracted in 23 (66%) patients, and bile stones were removed in 20 (57%) patients. Casts (*p* = 0.01) and bile stones (*p* = 0.04) were significantly more often extracted in those patients who only underwent endoscopy, but no further multidisciplinary treatment, as shown in Table 2.

In most patients, plastic stents were replaced every 1–3 months until NAS was resolved. On average, stents were changed after 2.3 months (ITBL: 2.7 months; IBL: 1.5 months). In 3 patients, self-expanding metal-stents (SEMS) were placed.

A total of 29 (83%) patients had an additional anastomotic stricture, which was resolved in 27 (93%) cases during the endoscopic stenting program. Of the remaining two patients, one patient died due to cholangitis and liver abscesses, and one patient was still undergoing the stent program at the end of follow-up.

Furthermore, 16 (46%) patients terminated the ERCP-based stent program successfully (ITBL: 13, 46%; IBL: 3, 43%). The median duration of the ERCP-based stent program was 34 months, with a median of 14 performed ERCPs. Furthermore, insertion of stents increasing in size and/or number was the aim, with the insertion of up to four stents at one time.

Five (14%) patients underwent a subsequent surgical approach with a bilioenteric anastomosis. All these had either extrahepatic or combined strictures of the biliary system.

At the end of follow-up, 21 (60%) patients were still alive, nine (26%) died due to cholangitis (ITBL: 6, 21%; IBL: 3, 43%), one (3%) patient died during subsequent surgery after failure of the ERCP-based stent program, and four (11%) patients died due to causes not related to NAS after successful termination of the ERCP-based stent program. Of the nine patients who died during the period of the ERCP-based stent program, one (11%) patient had a solitary extrahepatic stricture, and eight patients had either a solitary intrahepatic stricture (3, 33%) or a combined extra- and intrahepatic stricture (5, 56%). However, no statistical significance was found between intrahepatic or extrahepatic strictures as shown in Table 2. Further data with reference to the ITBL and IBL subgroups, as well as the different locations of the strictures, are shown in Table 3 and Supplementary Tables S2 and S3.

We did not observe any statistical differences between ITBL and IBL with respect to mortality during the ERCP-based stent program (*p* = 0.1) or successful termination of the ERCP program (*p* = 0.7). Data are displayed in Supplementary Figure S2.

Patients with primary biliary cholangitis (PSC) represent a special patient group because they already have diffuse strictures as an underlying disease comparable to NAS. Three patients with PSC were represented in the present study. Two patients had an ITBL, and one patient had an IBL. In all three cases, the primary ERCP-based stent program failed. Two patients received surgical intervention by bilioenteric anastomosis, and one patient was further treated by PTBD. The one patient without procedural change had pure intrahepatic strictures and died due to cholangitis with multiorgan failure.

The most common complications during the ERCP-based stent program were cholangitis in 26 (74%) patients, mild post-ERCP pancreatitis during the entire stent program in eight (23%) patients, and bleeding in one (4%) patient. No patient died or had a perforation directly associated with the ERCP. All data on complications related to ERCPs are presented in Table 2 and Supplementary Table S3.

- Variables for termination of ERCP-based stent program and overall mortality

Regression analysis of ITBL vs. IBL and type of NAS for the primary endpoints successful termination of ERCP-based stent program and overall mortality showed no significant difference among the different types of strictures. Data are shown in Supplementary Table S4.

Furthermore, univariate analysis was performed for the primary endpoints. No patient or surgical variables or laboratory values had an impact on successful termination of the ERCP-based stent program.

Overall mortality was associated with increased age (HR: 1.13, 95% CI: 1.01–1.26; *p* < 0.05), bilirubin (log-transformed, HR: 5.22 95% CI; 2.22–12.31; *p* < 0.05), alanine aminotransferase (log-transformed, HR: 1.97, 95% CI: 1.22–3.19; *p* < 0.05), and aspartate aminotransferase (log-transformed, HR: 2.77, 95% CI 1.60–4.80; *p* < 0.05). Complete data of the Cox proportional hazards regression analyses are presented in Table 4.

## 4. Discussion

Data on NAS and its therapy are still scant in the literature, even though NAS occurs in up to 30% of patients following orthotropic liver transplantation [1,10,18,19,20,21,22,23,24]. The present study is, to the best of our knowledge, one of the largest trials with a focus on the treatment of NAS, and it is the first to evaluate NAS in both its subtypes (ITBL and IBL) for all three different types of strictures in the bile system. Furthermore, data on the treatment of IBL are currently unavailable in the literature. In comparison to published trials, the present one focused not only on single interventions such as the endoscopic treatment but also on consecutive procedures. Accordingly, the limitations of all available treatment options were evaluated by this trial.

While IBL is less common than ITBL, the overall mortality, success rate of the ERCP-based stent program, and switch to a surgical approach did not differ significantly between the two. However, the present data might be underpowered to detect differences in IBL and ITBL. Despite a different pathogenesis leading to strictures in IBL and in ITBL, both appear alike on cholangiography [25]. Therefore, treatment strategies are likely similar.

Our data confirm that strictures can be resolved in about 50% of cases by repeat endoscopic stenting. Other studies found a slightly higher success rate of up to 66% [10,26]. This may be due to differing definitions of successful treatment. In the case of biliary strictures, current literature and guidelines suggest repeat stenting for about 1 year [27,28,29]. However, endoscopic stenting may need to be repeated noticeably more often in individual cases with NAS due to multiple and complex strictures in the bile duct system. In comparison, anastomotic strictures were resolved in 93% of all cases. The present study based on real-world data highlights that NAS, especially with intrahepatic strictures, is still a severe problem with relevant mortality. Our data suggest that extensive removal of stones and casts represents a contributory factor to successful stenting. Therefore, the necessity of additional multidisciplinary treatment approaches might be reduced. Nevertheless, bilioenteric anastomosis and PTBD can help to resolve NAS strictures after failure of the endoscopic stenting.

**Figure 2:**
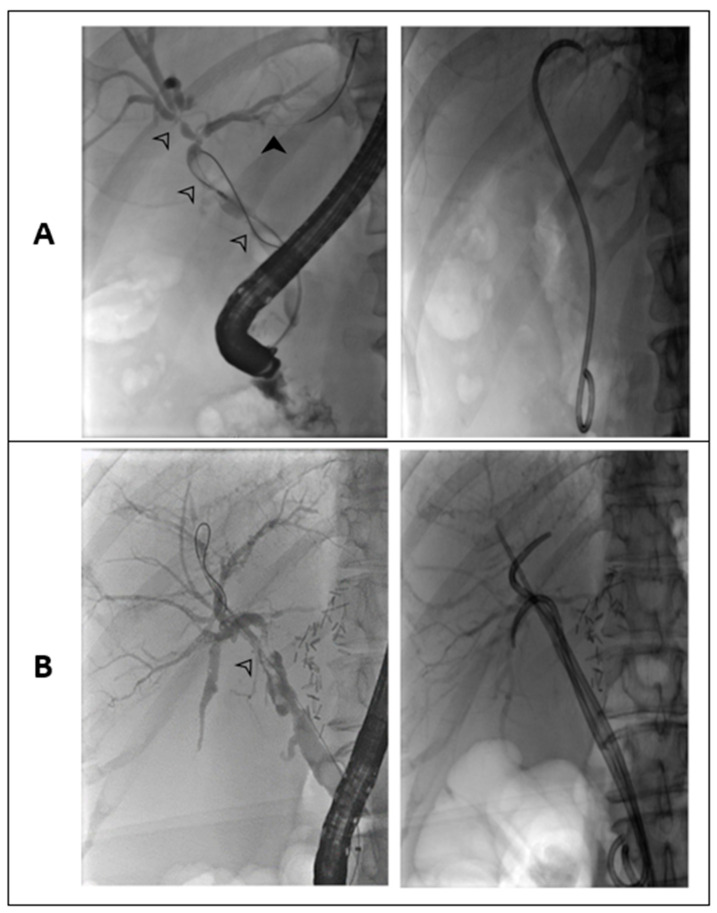
Fluoroscopic images of two different patients undergoing ERCP with indication NAS. (**A**) Type III strictures with two extrahepatic strictures and one in the hilar region (white arrowheads). These strictures were treated by a plastic stent. However, a peripheral bile duct remains narrowed and cannot be reached by stenting (black arrow). (**B**) Dominant extrahepatic stricture (type I) reaching the hilar region (white arrow). A total of three stents were placed in the common bile duct.

Compared to intrahepatic strictures, treatment of extrahepatic ones had a trend toward higher success of the stent program, a better outcome of bilioenteric anastomosis, and a lower mortality. Reasons might be that intrahepatic strictures beyond the hilar region are usually neither reachable with stents nor resolved with a bilioenteric anastomosis. Figure 2 highlights this problem in an example of two patients with NAS in our stent program.

Only PTBD is a reasonable option in intrahepatic strictures, but it is limited to a single bile duct. Therefore, balloon dilatation might be a solution for multiple intrahepatic strictures. Balloon dilatation of biliary anastomotic strictures alone was compared to dilatation plus stenting and found to yield significantly better results [21]. As stenting might promote ascending bile duct infections, another advantage of balloon dilatation might be a reduced rate of cholangitis. However, balloon dilatation has limitations in cases of long strictures and bile duct junctions [30].

Elevated bilirubin and transaminase levels were shown to be risk factors for higher mortality of NAS. No good predictive marker for successful termination of the ERCP-based stent program could be found, as similarly shown by Graziadei et al. [10]. In their patients with anastomotic strictures, cholestasis parameters normalized after successful completion of the ERCP-based stent program. However, in NAS, cholestasis parameters remained elevated even after endoscopic resolution of significant strictures and clinical improvement. Therefore, successful treatment of NAS can currently only be assessed via ERCP.

Limitations of this study were due to its retrospective character. Nonetheless, compared to previous studies, patients’ characteristics and incidence of NAS appear representative [1,10,18,19,20,21,22,23,24,31]. Further limitations were the single-center design and the limited number of patients, especially in the subgroup analysis. Nevertheless, as a transplant center and tertiary referral center, the single center used for this study provides care for patients within a large geographic radius.

Patients with NAS and additional anastomotic stricture were included in the present study. This could have introduced bias, as it remains unclear which stricture led to the observed results. However, it seems reasonable that the effects of anastomotic strictures on the endpoints were negligible because almost all anastomotic strictures were resolved within 1 year, and no patient died during that time. Another limitation is that the ERCP-based stent program could have been terminated at the discretion of the endoscopist. However, patients underwent further clinical and laboratory follow-up with continuation of the stent program if indicated.

## 5. Conclusions

Primary endoscopic stenting followed by subsequent surgery with PTBD is the current standard of care in NAS treatment [2,27,28,29]. However, our data suggest that endoscopic and multidisciplinary approaches resolve NAS only in about half of the patients and are less effective in diffuse intrahepatic strictures compared to extrahepatic ones. Extensive removal of stones and casts could reduce the need for further measures.

Furthermore, no laboratory values and patient- or procedure-related characteristics were found that help to predict a successful termination of endoscopic stenting, while age, bilirubin, AST, and ALT were associated with increased mortality. Procedure-related mortality was low. Major adverse events during the ERCP-based stent program were cholangitis and mild pancreatitis, with cholangitis being the most important cause of death in the presence of NAS.

Further trials with expanded cohort sizes are needed to allow development of predictive models for treatment outcomes. Special focus should also be placed on the number of strictures in NAS and on further investigation of which intrahepatic strictures are resistant to endoscopic and multidisciplinary treatment, to predict the need for early re-transplantation to avoid cholangitis with multiorgan failure without further treatment options.

## Figures and Tables

**Table 1:** Baseline characteristics.

	ITBL (*n* = 28)	IBL (*n* = 7)	Total (*n* = 35)	*p*-Value
Liver disease before transplantation				n.s.
Viral, *n* (%)	15 (54%)	5 (71%)	19 (54%)	
Alcohol, *n* (%)	4 (14%)	0 (0%)	4 (11%)	
NASH, *n* (%)	1 (4%)	0 (0%)	1 (3%)	
Autoimmune, *n* (%)	4 (14%)	1 (14%)	5 (14%)	
PSC, *n* (%)	2 (7%)	1 (14%)	3 (9%)	
Acute liver failure, *n* (%)	2 (7%)	1 (14%)	3 (9%)	
Other, *n* (%)	3 (11%)	0 (0%)	3 (9%)	
Age (years) *	63 (53.25/67.25)	63 (55.5/64.5)	63 (54.5/66.5)	n.s.
Male sex, *n* (%)	21 (75%)	3 (43%)	24 (69%)	n.s.
MELD at liver transplantation *	15 (11.5/29)	19 (11.5/33.5)	16.5 (11.25/29.5)	n.s.
Hypertension, *n* (%)	14 (50%)	4 (57%)	18 (51%)	n.s.
Heart disease, *n* (%)	4 (14%)	0 (0%)	4 (11%)	n.s.
Arteriosclerosis, *n* (%)	4 (14%)	0 (0%)	4 (11%)	n.s.
Diabetes mellitus, *n* (%)	10 (36%)	1 (14%)	11 (31%)	n.s.
Kidney disease, *n* (%)	13 (46%)	3 (43%)	16 (46%)	n.s.
CMV reactivation, *n* (%)	8 (29%)	3 (43%)	11 (31%)	n.s.
Cold ischemic time (h:min) *	8:41 (7:56/10:23)	9:52 (7:36/10:57)	8:45 (7:54/10:47)	n.s.
Warm ischemic time (min) *	43 (36/47)	52 (45/57)	45 (36/49)	n.s.
Surgical revision of the bile duct anastomosis, *n* (%)	4 (14%)	1 (14%)	5 (14%)	n.s.
Surgical revision of the artery, *n* (%)	(0%)	7 (100%)	7 (24%)	<0.001

* Data are presented as the median (25th/75th percentile) unless indicated otherwise. n.s.: not significant.

**Table 2:** Analysis of all 30 patients who finished the ERCP-based stent program or multidisciplinary therapy. Those five patients who were still part of endoscopic stenting were excluded.

Variables	Only Endoscopic Therapeutic Approach		Multidisciplinary Therapy		*p*-Value of All Four Values	p-Value of Endoscopic Therapy Only	HR (95% CI) of Endoscopic Therapy Only
	Alive (*n* = 16)	Dead (*n* = 9)	Alive (*n* = 4)	Dead (*n* = 1)			
ITBL, *n* (%)	13 (81%)	6 (67%)	3 (75%)	1 (100%)	n.s.	n.s.	0.46 (0.71–2.99)
NAS locus intrahepatic, *n* (%)	10 (62%)	8 (89%)	2 (50%)	1 (100%)	n.s.	n.s.	0.21 (0.21–2.10)
Male sex, *n* (%)	9 (56%)	7 (77%)	4 (100%)	1 (100%)	n.s.	n.s.	2.72 (0.43–17.4)
Age *	64 (57/68)	63 (42/65)	63 (47/72)	51	n.s.	n.s.	n.a.
MELD *	15 (10/31)	19 (10/29)	13 (9/26)	40	n.s.	n.s.	n.a.
Hypertension, *n* (%)	11 (69%)	3 (33%)	3 (75%)	0 (0%)	n.s.	n.s.	4.40 (0.77–25.1)
Heart disease, *n* (%)	2 (13%)	1 (11%)	1 (25%)	0 (0%)	n.s.	n.s.	1.14 (0.09–14.7)
Atherosclerosis, *n* (%)	2 (13%)	0 (0%)	1 (25%)	0 (0%)	n.s.	n.s.	8.00 (0.80–79.7)
Diabetes mellitus, *n* (%)	5 (31%)	3 (33%)	1 (25%)	0 (0%)	n.s.	n.s.	0.91 (0.16–5.20)
Kidney disease, *n* (%)	10 (63%)	3 (33%)	1 (25%)	0 (0%)	n.s.	n.s.	3.33 (0.60–18.5)
CMV infection, *n* (%)	5 (31%)	3 (33%)	1 (25%)	0 (0%)	n.s.	n.s.	0.93 (0.15–5.61)
Cold ischemic time in h *	8 (7/11)	8 (7/10)	9 (7/12)	10	n.s.	n.s.	n.a.
Warm ischemic time in min *	44 (41/48)	48 (44/51)	40 (33/48)	45	n.s.	n.s.	n.a.
Median duration until diagnosis in months *	6 (3/19)	5 (3/15)	1 (1/3)	7	0.04	0.05	n.a.
Median duration of stent program in months *	19 (12/44)	14 (4/59)	41 (9/48)	41	0.02	n.s.	n.a.
Number of ERCPs since diagnosis of NAS *	13 (9/14)	11 (6/26)	13 (0/31)	29	0.06	n.s.	n.a.
Number of ERCPs in total *	13 (9/15)	13 (7/31)	13 (0/31)	30	0.05	n.s.	n.a.
Cast extraction, *n* (%)	13 (81%)	7 (78%)	0 (0%)	1 (100%)	0.01	n.s.	1.24 (0.17–9.25)
Bile stone extraction, *n* (%)	7 (78%)	11 (69%)	0 (0%)	1 (100%)	0.04	n.s.	0.63 (0.10–4.18)
Balloon dilatation, *n* (%)	6 (38%)	5 (56%)	1 (25%)	0 (0%)	n.s.	n.s.	0.48 (0.09–2.52)
Cholangitis, *n* (%)	14 (88%)	6 (67%)	3 (25%)	0 (0%)	n.s.	n.s.	3.50 (0.46–26.6)
Pancreatitis, *n* (%)	6 (38%)	0 (0%)	1 (25%)	0 (0%)	n.s.	0.07	n.a.
Bleeding after ERCP, *n* (%)	1 (6%)	0 (0%)	0 (0%)	0 (0%)	n.s.	n.s.	n.a.
Perforation, *n* (%)	0 (0%)	0 (0%)	0 (0%)	0 (0%)	n.s.	n.s.	n.a.

* Data are presented as tge median (25th/75th percentile) unless indicated otherwise; n.a.: not applicable, n.s.: not significant.

**Table 3:** Overview of procedure-related data and outcome analysis of NAS, ITBL, and IBL, as well as subgroup analysis of stricture types (type 1—extrahepatic strictures/type 2—intrahepatic strictures/type 3—combined strictures).

		ITBL				IBL				Total	*p*-Value
		Extrahepatic Strictures	Intrahepatic Strictures	Combined Strictures	Total ITBL	Extrahepatic Strictures	Intrahepatic Strictures	Combined Strictures	Total IBL		Comparison ITBL vs. IBL
Number		8 (29%)	6 (21%)	14 (50%)	28 (80%)	2 (29%)	1 (14%)	4 (57%)	7 (20%)	35 (100%)	
Procedure-related data	Successful termination of ERCP-based stent program	4 (50%)	2 (33%)	7 (50%)	13 (46%)	2 (100%)	0 (0%)	1 (25%)	3 (43%)	16 (46%)	n.s.
	Still part of ERCP-based stent program	1 (13%)	2 (33%)	2 (14%)	5 (18%)	0 (0%)	0 (0%)	0 (0%)	0 (0%)	5 (14%)	n.s.
	Switch to surgical approach	2 (25%)	0 (0%)	2 (14%)	4 (14%)	0 (0%)	0 (0%)	1 (25%)	1 (14%)	5 (14%)	n.s.
Outcome analysis	Alive at end of follow-up period	6 (75%)	2 (33%)	9 (64%)	17 (61%)	2 (100%)	0 (0%)	2 (50%)	4 (57%)	21 (60%)	n.s.
	Deaths during the ERCP-based stent program	1 (13%)	2 (33%)	3 (21%)	6 (21%)	0 (0%)	1 (100%)	2 (50%)	3 (43%)	9 (26%)	n.s.
	Death after surgical approach	0 (0%)	0 (0%)	1 (7%)	1 (4%)	0 (0%)	0 (0%)	0 (0%)	0 (0%)	1 (3%)	n.s.
	Cause of death other than NAS	1 (13%)	2 (33%)	1 (7%)	4 (14%)	0 (0%)	0 (0%)	0 (0%)	0 (0%)	4 (11%)	n.s.

Overview of all 35 patients with NAS in the ERCP-based stent program. All five patients with primary surgical approach or lost to follow-up were excluded (ITBL: 4, IBL: 1). All data are presented as absolute numbers (*n*) and percentages (%) based on the number of patients in each subgroup. IBL: ischemic biliary lesion, ITBL: ischemic-type biliary lesion. n.s.: not significant.

**Table 4:** Cox proportional hazards regression analyses of baseline characteristics, surgical characteristics, and laboratory values in relation to overall mortality and termination of the ERCP-based stent program.

		Overall Mortality		Successful Termination of the ERCP-Based Stent Program	
		HR (95% CI)	*p*-Value	HR (95% CI)	*p*-Value
Patient characteristics	Age	1.13 (1.01–1.26)	0.03	0.99 (0.94–1.05)	n.s.
	Male sex	2.38 (0.92–1.09)	n.s.	1.16 (0.44–3.07)	n.s.
	MELD	1.00 (0.91–1.10)	n.s.	1.03 (0.98–1.07)	n.s.
	Hypertension	0.13 (0.01–1.46)	n.s.	0.68 (0.25–1.85)	n.s.
	Heart disease	47.7 (0.85–2680)	0.06	0.24 (0.03–1.81)	n.s.
	Arteriosclerosis	2.01 (0.31–129)	n.s.	0.67 (0.15–2.97)	n.s.
	Diabetes mellitus	14.2 (0.77–261)	0.08	0.37 (0.12–1.16)	n.s.
	Kidney disease	0.23 (0.19–2.76)	n.s.	0.64 (0.23–1.81)	n.s.
	CMV infection	0.52 (0.11–2.50)	n.s.	0.42 (0.13–1.33)	n.s.
Surgical characteristics (two-sided *p*-values)	Surgical revision of the bile duct anastomosis	0.53 (0.06–4.58)	n.s.	0.60 (0.08–4.76)	n.s.
	Surgical revision of the arterial anastomosis	4.62 (0.94–22.69)	0.06	1.53 (0.20–11.8)	n.s.
	Cold ischemic time	0.99 (0.99–1.004)	n.s.	0.99 (0.99–1.003)	n.s.
	Warm ischemic time	0.99 (0.97–1.03)	n.s.	1.01 (0.99–1.03)	n.s.
Laboratory values (one-sided *p*-values)	log (Bilirubin)	5.22 (2.22–12.31)	<0.0001	1.61 (0.87–2.97)	n.s.
	log (ALT)	1.97 (1.22–3.19)	0.006	0.58 (0.30–1.15)	n.s.
	log (AST)	2.77 (1.60–4.80)	0.0003	0.33 (0.10–1.09)	n.s.
	log (GGT)	0.50 (0.26–0.96)	n.s.	0.54 (0.33–0.90)	n.s.
	log (AP)	0.33 (0.10–1.16)	n.s.	0.39 (0.17–0.85)	n.s.

ALT: alanine aminotransferase, AP: alkaline phosphatase, AST: aspartate aminotransferase, CI: 95% confidence interval, GGT: gamma-glutamyl transferase, HR: hazard ratio, MELD: model of end-stage liver disease, n.s.: not significant.

## Data Availability

The data presented in this study are available on request from the corresponding author. The data are not publicly available for the protection of privacy.

## Supplementary Materials

The following supporting information can be downloaded at https://www.mdpi.com/article/10.3390/jcm12103491/s1: Figures S1: Overview of the treatment procedure and outcome of all patients identified with NAS from 2008 to 2016 at the Frankfurt University Hospital. Figure S2: Survival analysis using Kaplan-Meier survival analysis on mortality during ERCP-based stent program (A) and Cox regression analysis on successful termination of ERCP-based stent program (B). No statistical significance was observed between ITBL and IBL in respect to mortality during ERCP-based stent program (*p* = 0.1), or successful termination of the ERCP program (*p* = 0.7). Table S1: Data of the nine deceased patients during the ERCP-based stent program. Table S2: Number of ERCPs and duration of the ERCP-based stent program. Table S3: Procedures and complications during ERCP-based stent program. Table S4: Regression analysis of ITBL vs IBL and type of NAS regarding successful termination of ERCP-based stent program and overall survival.
