# Supplementary material for: Treatment of Non-Anastomotic Biliary Strictures after Liver Transplantation: How Effective Is Our Current Treatment Strategy?
Source: J Clin Med. 2023 May 16;12(10):3491. doi: 10.3390/jcm12103491 (PMC10219240; doi:10.3390/jcm12103491)
Supplement: Supplementary file 1 [file jcm-12-03491-s001.zip › jcm-2358366-supplementary.pdf]

Journal of
Clinical Medicine

MDPI

Supplementary Materials:

The following supporting information can be downloaded at: https://www.mdpi.com/article/10.3390/jcm12103491/s1, Figueres S1-S2 and Table S1-S4

Supplementary Figure S1

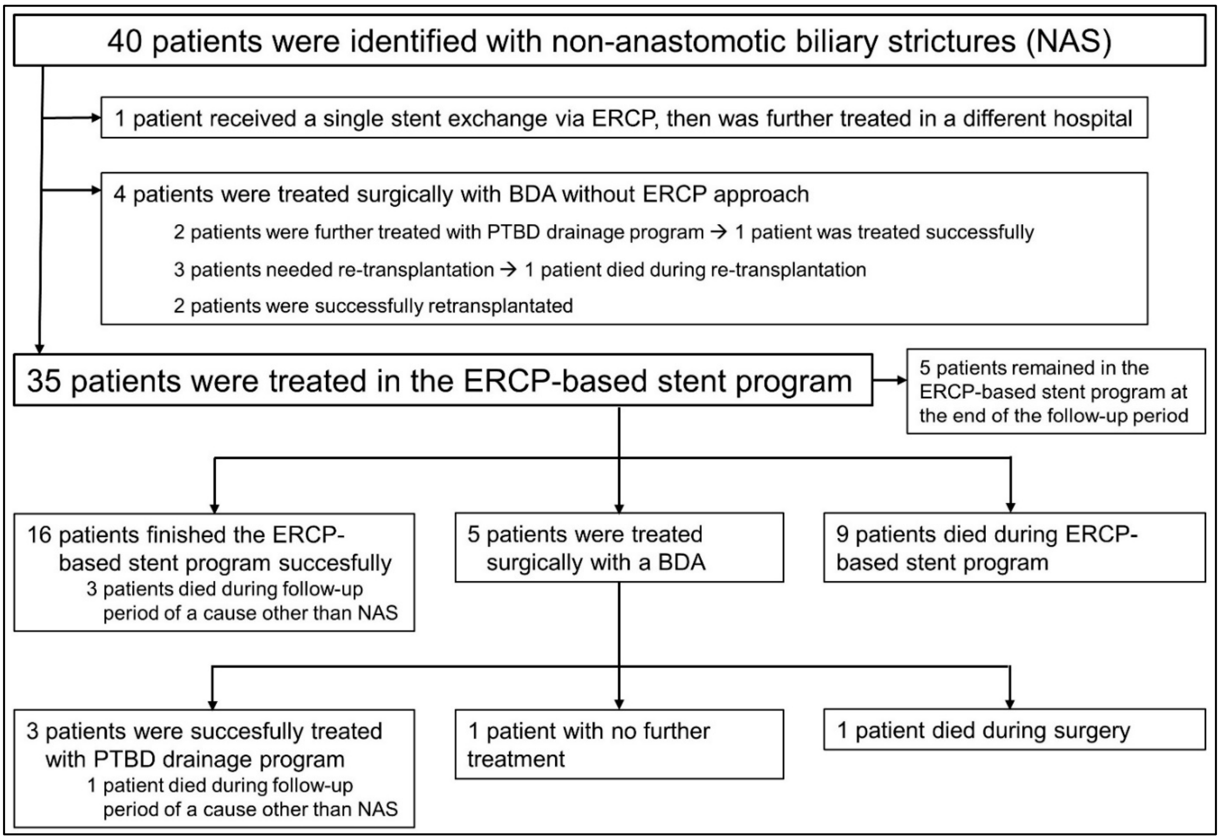

Figure S1: Overview of the treatment procedure and outcome of all patients identified with NAS from 2008 to 2016 at the Frankfurt University Hospital. BDA: bilioenteric anastomosis; ERCP: Endoscopic retrograde cholangiopancreaticography; IBL: ischemic biliary lesion; ITBL: ischemic-type biliary lesion; PTBD: percutaneous transhepatic biliary drainage

Supplementary Figure S2

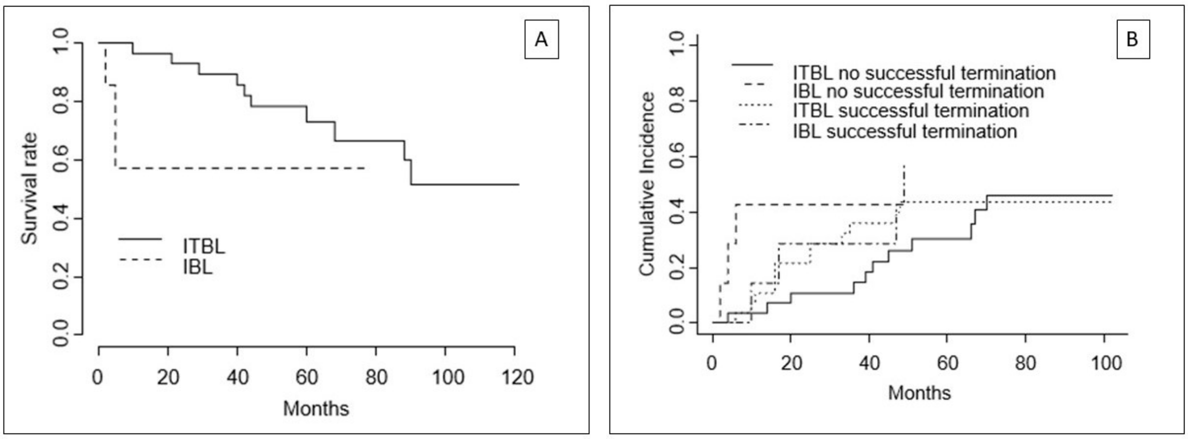

Figure S2: Survival analysis using Kaplan-Meier survival analysis on mortality during ERCP-based stent program (A) and Cox regression analysis on successful termination of ERCP-based stent program (B). No statistical significance was observed between ITBL and IBL in respect to mortality during ERCP-based stent program (p=0.1), or successful termination of the ERCP program (p=0.7).

Supplementary Table S1: Data of the nine deceased patients during the ERCP-based stent program

Patient	1	2	3	4	5	6	7	8	9
ITBL or IBL	ITBL	IBL	ITBL	IBL	ITBL	IBL	ITBL	ITBL	ITBL
NAS type	I	II	II	III	III	III	II	III	III
Age at death	69	66	58	44	63	62	31	39	64
Gender	Male	Male	Male	Female	Male	Male	Female	Male	Male
Reasons for cirrhosis/transplantation	Alcohol and HCC	Viral and HCC	PSC	Acute liver failure	Viral	Vial and HCC	Autoimmune-hepatitis	Viral	Viral
MELD at death	15	24	34	39	28	40	40	39	38
Cause of death	Cholangitis and nosocomial pneumonia	Cholangitis with hepatic abscesses and pleural empyema	Cholangitis and spontaneous bacterial peritonitis	Cholangitis with hepatic abscesses	Cholangitis and nosocomial pneumonia	Cholangitis with hepatic abscesses	Cholangitis and nosocomial pneumonia	Cholangitis	Septic shock due to cholangitis
Contraindication of re-transplantation	Critical illness myopathy	Patient decision	Non transplantable during septic shock	No timely organ offer	Combined aortic stenosis II° and insufficiency III°	Non transplantable during septic shock	No timely organ offer	Centre decision because of rejection of blood products by the patient	Non transplantable during septic shock
Duration from transplantation until diagnosis of NAS in months	10	2	45	1	3	5	18	3	12
Duration of stent program in months	67	2	51	4	4	6	39	14	70
Number of ERCPs since diagnosis of NAS	32	4	13	7	5	6	20	11	39
Number of ERCPs in total	43	6	18	7	7	8	20	13	51

ERCP: endoscopic retrograde cholangiopancreatography; HCC: hepatocellular carcinoma; IBL: ischemic biliary lesion; ITBL: ischemic-type biliary lesion; NAS: Non-anastomotic biliary strictures

Supplementary Table S2: Number of ERCPs and duration of the ERCP-based stent program

	ITBL				IBL				Total
	Extrahepatic strictures	Intrahepatic strictures	Combined strictures	Total ITBL	Extrahepatic strictures	Intrahepatic strictures	Combined strictures	Total IBL	
Number	8 (29%)	6 (21%)	14 (50%)	28 (80%)	2 (29%)	1 (14%)	4 (57%)	7 (20%)	35 (100%)
Median duration until diagnosis in months	4 (2/8.5)	6 (5.25/15)	7 (3/12)	6 (3/11.5)	8; 52	2	2 (1/5)	3 (1/5)	5 (2.25/9.75)
Median duration of stent program in months	32.5 (7.25/64.25)	44 (32.5/63.75)	29.5 (15.5/47.25)	35.5 (16/50.5)	47; 17	4	8 (4.5/39.25)	10 (4/47)	34 (11/49)
Number of ERCPs since diagnosis of NAS	19 (8/29)	26 (13/37)	14 (12/24)	15 (12/30)	13; 9	4	7 (2/9)	7 (4/9)	13 (9/26)
Number of ERCPs in total	21 (10/31)	26 (17/38)	14 (11/25)	17 (11/31)	13; 9	6	8 (2/9)	8 (6/9)	14 (9/26)

Data presented as median (25. /75. percentile).

ERCP: endoscopic retrograde cholangiopancreatography; IBL: ischemic biliary lesion; ITBL: ischemic-type biliary lesion; NAS: non-anastomotic stricture.

Supplementary Table S3: Procedures and complications during ERCP-based stent program

	ITBL				IBL				Total
	Extrahepatic strictures	Intrahepatic strictures	Combined strictures	Total ITBL	Extrahepatic strictures	Intrahepatic strictures	Combined strictures	Total IBL	
Number	8 (29%)	6 (21%)	14 (50%)	28 (80%)	2 (29%)	1 (14%)	4 (57%)	7 (20%)	35 (100%)
Cast extraction, n (%)	3 (38%)	5 (83%)	11 (79%)	19 (68%)	2 (100%)	0 (0%)	2 (50%)	4 (57%)	23 (66%)
Bile stone extraction, n (%)	2 (25%)	5 (83%)	9 (64%)	16 (57%)	2 (100%)	1 (100%)	1 (25%)	4 (57%)	20 (57%)
Balloon dilatation, n (%)	3 (38%)	5 (93%)	6 (43%)	14 (50%)	0 (0%)	1 (100%)	0 (0%)	1 (14%)	15 (43%)
Stenting, n (%)	8 (100%)	6 (100%)	14 (100%)	28 (100%)	2 (100%)	1 (100%)	4 (100%)	7 (100%)	35 (100%)
Cholangitis, n (%)	6 (75%)	6 (100%)	9 (64%)	21 (75%)	2 (100%)	0 (0%)	3 (75%)	5 (71%)	26 (74%)
Pancreatitis, n (%)	1 (13%)	0 (0%)	6 (43%)	7 (25%)	0 (0%)	0 (0%)	1 (25%)	1 (14%)	8 (23%)
Bleeding after ERCP, n (%)	0 (0%)	1 (17%)	0 (0%)	1 (4%)	0 (0%)	0 (0%)	0 (0%)	0 (0%)	1 (3%)
Perforation, n (%)	0 (0%)	0 (0%)	0 (0%)	0 (%)	0 (0%)	0 (0%)	0 (0%)	0 (0%)	0 (0%)
Procedure related mortality, n (%)	0 (0%)	0 (0%)	0 (0%)	0 (%)	0 (0%)	0 (0%)	0 (0%)	0 (0%)	0 (0%)

ERCP: Endoscopic retrograde cholangiopancreatography; IBL: ischemic biliary lesion; ITBL: ischemic-type biliary lesion

Supplementary Table S4: Regression analysis of ITBL vs IBL and type of NAS regarding successful termination of ERCP-based stent program and overall survival

	Overall mortality		Termination of the ERCP-based stent program	
	HR (95%-CI)	p-value	HR (95%-CI)	p-value
ITBL vs IBL	0.86 (0.16-4.55)	n.s.	0.57 (0.10-3.19)	n.s.
Type I vs II + III	5.26 (0.85-33.3)	n.s.	0.30 (0.04-2.29)	n.s.
Type II + III vs I	3.69 (0.65-54)	n.s.	0.58 (0.06-17.55)	n.s.
Type III vs I + II	1.00 (0.23-3.52)	n.s.	1.71 (0.25-11.78)	n.s.

CI: confidence interval; ERCP: endoscopic retrograde cholangiopancreatography; IBL: ischemic biliary lesion; ITBL: ischemic-type biliary lesion; HR: hazard ratio; Type I: extrahepatic strictures; type II: intrahepatic strictures; type III: extra- and intrahepatic stricture.
